# New York Letter

**Published:** 1895-09

**Authors:** 


### NEW YORK LETTER.

NEW YORK, August 31, 1895.

The medical world has sustained a loss in the death of Dr. Wm. C. Jarvis, which occurred July 30th, at Willet's Point, New York. He was professor of diseases of the throat in the University of the City of New York, a position to which he was appointed at the age of twenty-six. His name is widely known from the ingenious and useful throat and nose instruments which bear his name. He was only forty years of age at his death.

Following the robbery, last month, of Dr. Drury, of Brooklyn, many Brooklyn physicians refused calls coming to them from strange sources, and the superintendent of police offered the services of the department to provide an escort to those desiring it, when going to what they believed to be a suspicious call. It is not known whether any physician has availed himself of this generous offer.

The State Board of Health has found the existence of anthrax among the cattle in many portions of the State.

A thoroughly up-to-date metropolitan daily, which strives to furnish all the reading matter for the household, having its children's column, its woman's column, its daily novel, its pseudo-scientific column, and a multitude of other columns, in addition to its inaccurate news columns, has for some time also endeavored to usurp the place of the family doctor and furnish its readers with free medical advice under the title of "Talks with the Doctor." The brilliant and undoubtedly deserving, though practiceless disciple of Æsculapius who presides over the department, deals out prescriptions with a free hand to correspondents seeking advice. The *Medical Record* of August 3d protests (though we fear 'tis in vain) against this as a dangerous thing and far worse even than counter prescribing by the drug clerk. Our learned brother advised one correspondent, who asked how to take bromide for a "nervous" headache, to take fifteen or thirty grains every two or three hours. It would be interesting to know, in case of suit for malpractice (a very remote possibility), whether the newspaper or the doctor could be held responsible.

The *Medical Record* of August 24th, page 260, contains an abstract entitled "Gonorrhœa, When Cured?" which states that the author, as a test whether a patient is cured, makes him drink a quart and a half of beer and then injects a two per cent solution of sublimate into the urethra. If the patient is cured no reaction follows, but, if not, a discharge will be set up within forty-eight hours. We imagine that if any one attempts to set up a urethritis by injecting a two per cent solution of bichloride, he will find that he has waked up the wrong man. It may be all right, but we do not think we should care to be the first to follow this advice, and we cannot help thinking that the abstracter has misread the figures.

Therapeutic suggestions in the treatment of gonorrhœa are always of interest, especially when backed up by good reasoning and tangible results. A therapeutic hint in the *Medical Record* of June 1st, an extract from a lecture by Dr. Rautier, says, "When the gonorrhœa is in the acute stage, I would advise not to interfere. Tell the patient to wear a suspensory bandage, to take an alkaline bath every three days, and in about ten days afterward to come back, and then you will cure him." Dr. Rautier, after this, uses solutions of permanganate of potash. Dr. W. A. Davison, of Fort Benton, Montana, protests in the *Medical Record*, July 27, page 122, that this might do in great medical centers in France, and possibly even the medical metropolis of the United States, but the medical man in this section who prescribed such a course of treatment in his cases of acute gonorrhœa, would most assuredly treat very few cases. He urges earliest possible treatment with hot injections, and in this the writer agrees with him. Dr. D's plan is to use a hot borated solution in a fountain syringe, to which is attached a soft rubber catheter. He elevates the reservoir only two feet above patient; unfortunately, he does not state whether patient stands or lies down. The catheter, well oiled, is inserted one inch, and water allowed to flow till this is presumably clean, then the catheter is inserted further, and again the fluid allowed to flow, and so on until the deeper portion of the urethra is reached, and even when necessary, the catheter may be pushed past the cut-off muscle and the bladder irrigated; one and a half quarts of fluid are used and irrigations performed daily. He further has the patients use every six hours, a soothing sedative mucilaginous injection of not more than half a drachm. And in the subacute or chronic, uses the following preparation:

| | | |
|---|---|---|
| ℞. | Morph. Sulph....... | ʒiv. |
| | Zinci Acetat....... | gr i. |
| | Aquæ............. | ℥iv. to ℥vi. |
| M. | | |

A similar method has long been used in New York, using a solution of 1-20000 bichloride, and making daily injections of a quart or more.

For two years, the writer has been following the method advocated by Janet, of Paris, using hot solutions of permanganate of potash, using instead of the catheter and retrojections, as above, a simple, blunt-pointed glass nozzle, or the urethral nozzle which comes with the "Alpha" fountain syringe, so wide that it may block the meatus and prevent the outflow when desirable. The solution used is made as hot as the patient can comfortably bear, and as time goes on, may be made much hotter than at the beginning of treatment. The strength at first is 1-4000, gradually increasing to 1-1000. One to two quarts of fluid are used. This method has been pursued in a large venereal clinic with a very large number of cases of gonorrhœa in all stages. Six hundred cases at least have been thus treated. Care is always taken in giving a patient his first injection and only a short *seance* is given, as it is apt to be followed by faintness if prolonged; after he is used to it, two quarts of very hot water may be used without inconvenience. The patient stands; the injection is five feet above the level of the penis. The penis is grasped an inch away from the meatus and gently held between finger and thumb so as to close the urethra at this point, and the urethra in front of this is washed, then another inch and so on. The patient always urinates first into two glasses, and so long as the second glass remains clear, the anterior urethra alone is irrigated, but as soon as there appears cloudiness of the second glass, showing beginning posterior urethritis, after irrigating the anterior urethra, the nozzle is pressed against the meatus, the urethra allowed to fill, and soon the fluid passes back into the bladder. When the bladder is full, the patient urinates. If it is difficult to force fluid past the cut-off muscle, tell the patient to try to urinate, and it will then easily pass into the bladder, or if the patient takes a deep inspiration and expiration, it is sometimes accomplished. In some few cases, it requires much patience to do this, and sometimes is impossible. Sometimes, the permanganate does not work well; then, change to 1-2000 Ag $NO_3$ solution hot, or bichloride 1-20000. In two years, by following this method, the writer has seen only two cases of epididymitis develop while the patient was undergoing treatment. The writer has almost entirely given up the use of the balsams and sandalwood oil, and claims for this method: 1. Rapid lessening of discharge. 2. Lessening of *ardor urinæ*. 3. No complaints (very few cases) of chordee. 4. Fewer cases of posterior urethritis. 5. A short attack of posterior urethritis, when it does set in. 6. Very marked decrease in cases of epididymitis. 7. Absence of stricture formation. Writers on gonorrhœa do not lay enough stress on the difference in the amenability to treatment of the first attack of gonorrhœa and subsequent attacks.

---

The pilgrimage to Lourdes this year is the largest that has ever taken place. From fifteen thousand to twenty thousand ill and afflicted were received there every day. A great many reputed miracles have been reported; the cases are examined at Lourdes by a board of physicians, three of whom are English-speaking Protestants. A despatch in the *Sun* says that the reports of the pilgrimage were "in some features most apalling. The Orleans station in Paris was filled day and night by thousands of sufferers enduring indescribable agonies in the effort to reach the healing waters. Most of the time the air rang with shrieks of pain as the almost dying pilgrims were loaded or unloaded on the cars, which were packed to their utmost capacity. The scene one afternoon, when a special train from Germany, laden with six hundred invalids, many of them desperate cases, arrived for a change of cars, was enough to drive mad any spectator without the strongest nerves." The officials of the Orleans railway state that the returning trains have not nearly as many passengers requiring assistance as the departing trains.

